# Perineal Lipoma With Accessory Labioscrotal Fold and Penis-like Phallus in a Female Infant With Unilateral Renal Agenesis^☆^

**DOI:** 10.1016/j.urology.2014.03.033

**Published:** 2014-07

**Authors:** William Mifsud, Nikola Sambandan, Paul Humphries, Neil J. Sebire, Imran Mushtaq

**Affiliations:** a Department of Histopathology, Level 3, Camelia Botnar Laboratories, Great Ormond Street Hospital for Children, London, United Kingdom; b Department of Urology, Great Ormond Street Hospital for Children, London, United Kingdom; c Department of Radiology, Great Ormond Street Hospital for Children, London, United Kingdom

## Abstract

We present a case of a female 46,XX infant with a perineal lipoma within an accessory labioscrotal fold containing a penis-like phallus, associated with contralateral renal agenesis and complete absence of Müllerian/paramesonephric structures. To our knowledge, this is the first report of perineal lipoma and accessory labioscrotal fold associated with urogenital abnormalities in a female. The case also has an exceptional penis-like phallus in the absence of Y chromosome material or evidence of virilization.

## Case Report

An 18-month-old infant was referred from another country to our hospital with ambiguous genitalia and absent right kidney. She was conceived by in vitro fertilisation to nonconsanguineous parents and there was no other significant family or medical history. There was no history of maternal illness, drug use, or radiation exposure during the pregnancy. After an uncomplicated antenatal course, she delivered a female infant of 2.9 kg by caesarean section at term.

**Figure 1:**
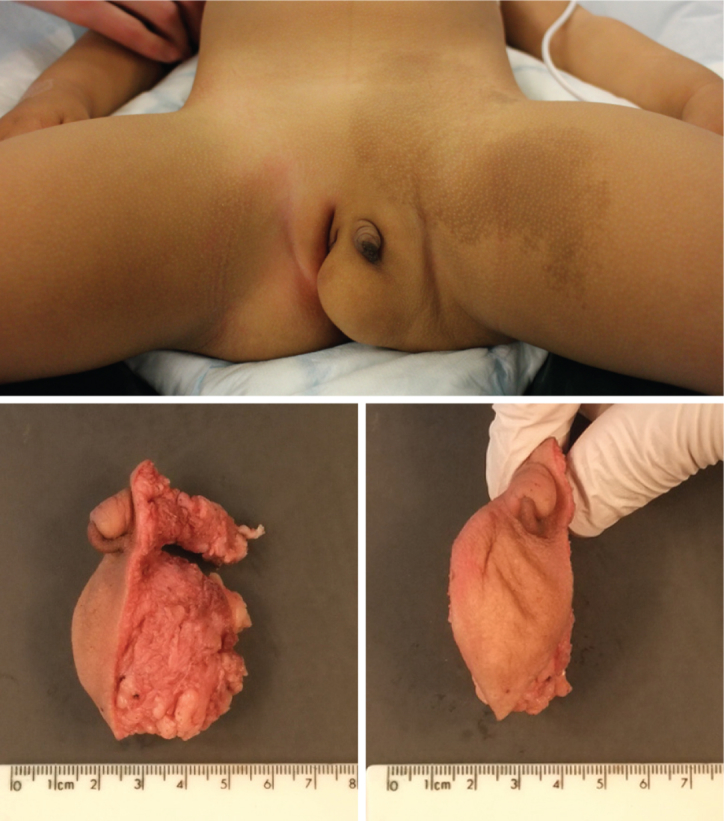
Top panel: photograph taken at perineal examination under anesthesia, showing left labial mass with an accessory phallus. Bottom panels: macroscopic photographs of the surgically excised specimen showing the accessory phallus extending above and beneath the skin, which is coarsely rugated and overlies a fatty mass. (Color version available online.)

Examination demonstrated a 5.0 × 2.8-cm left labial mass, which extended posteriorly to the left anal verge, with accessory phallus measuring 1.4 cm in length and 1.1 cm in width (Fig. 1). Adjacent were normal labia, clitoris, and urethral opening. There was no scrotum or palpable testis. Endoscopic examination confirmed a single opening in the perineum, which led into a short, wide urethra and normal sized bladder. There was also an accessory blind-ending urethra originating from the main urethra and extending toward the base of the accessory phallus. There was no vagina. There was a 20-cm flat pigmented lesion in the left inguinal region (Fig. 1). Physical examination was otherwise unremarkable.

**Figure 2:**
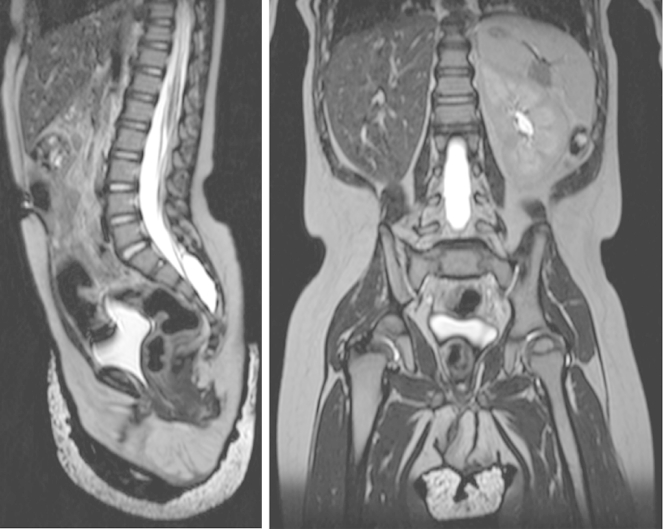
Sagittal (left) and coronal (right) T_2_-weighted magnetic resonance imaging views of the abdomen and pelvis, showing right renal agenesis and complete absence of Müllerian/paramesonephric duct structures.

Ultrasound and magnetic resonance imaging confirmed an absent right kidney with no Müllerian/paramesonephric duct structures (Fig. 2). Both ovaries were present but ectopic; the right ovary was located beneath the liver and the left ovary at the left pelvic inlet. The cortisol profile was normal, and there was a normal response to low-dose synacthen. Testosterone was 0.9 nmol/L at 3 weeks of age. A 3-day human chorionic gonadotrophin test at 18 months of age showed a normal female response, with undetectable androgen levels before and after stimulation. Sodium and other electrolytes were normal on repeated testing throughout the first 18 months of life. The karyotype was normal 46,XX from both blood and skin. Array comparative genomic hybridisation on the skin sample showed no Y chromosome-derived material. Laparoscopic examination confirmed the presence of ectopic ovaries, absence of the right kidney and Müllerian/paramesonephric duct structures. The ovaries were not biopsied.

She underwent genital reconstruction with excision of the labial mass and accessory phallus. The labial mass was lipomatous and excised through an elliptical incision. The accessory phallus had a well-developed corporal body and neurovascular bundle, which was traced proximally to its insertion into the left pubic ramus and detached.

Macroscopic examination of the excised mass (Fig. 1) showed skin with coarse rugae and deep furrows, suggestive of an accessory labioscrotal fold, overlying a fatty subcutaneous mass. The rugae terminated approximately 0.7 cm from the left long edge of the excised specimen. The accessory phallus extended 1.2 cm above the skin surface and 2.4 cm beneath the skin; it did not have a meatus, and the external part was anchored to the skin by a raphe extending to the tip. There was no structure reminiscent of a penile glans.

**Figure 3:**
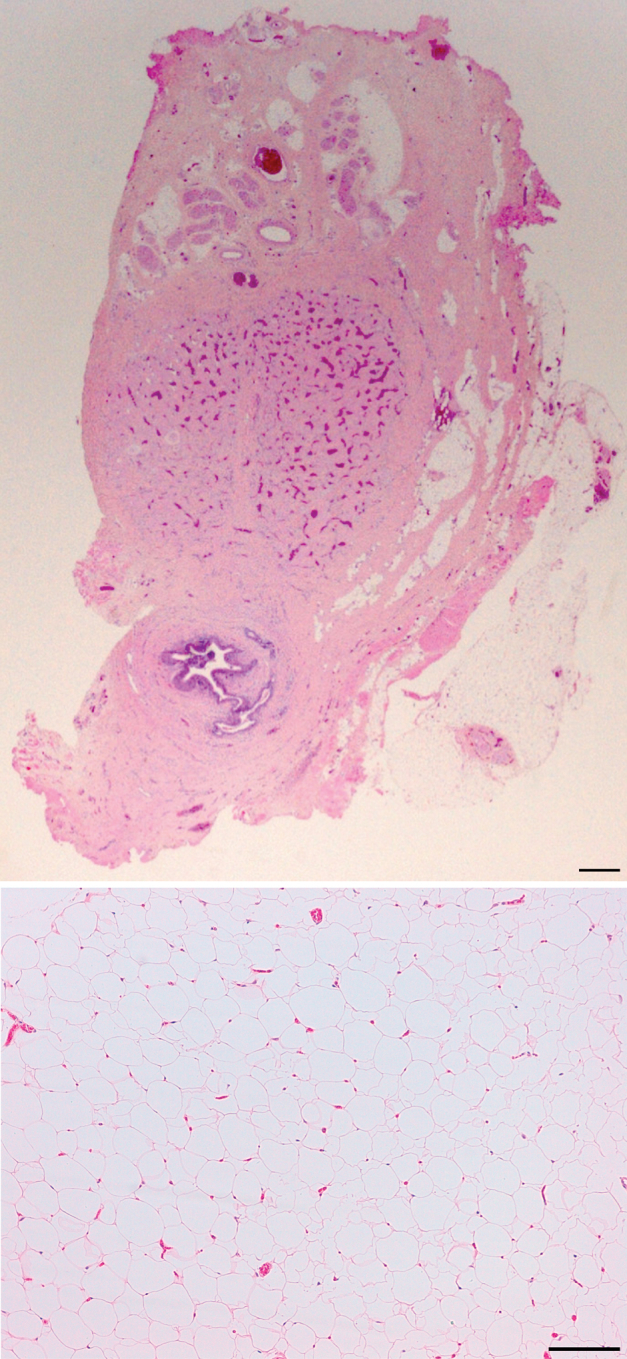
Top panel: photomicrograph of a transverse section through the subcutaneous portion of the penis-like phallus, demonstrating urethra surrounded by corpus spongiosum, paired corpora cavernosa separated by fibrous septum, dorsal neurovascular bundle, and fibrous tissue analogous to penile deep fascia. Bottom panel: photomicrograph of a transverse section through the perineal mass, showing a lipoma, with mature white adipocytes and numerous capillary blood vessels. There are no immature or atypical adipocytes, and there are no mitotic figures. Scale bars: 150 μm. (Color version available online.)

Microscopic examination of the phallus (Fig. 3) demonstrated a urethra lined by urothelium and surrounded by corpus spongiosum. Dorsal to these there were 2 corpora cavernosa separated by a fibrous septum, and a central, dorsal vein flanked by arteries. These structures were in turn surrounded by additional fibrous tissue. The appearances were reminiscent of penile histology. The fatty mass contained lobulated, mature white adipose tissue, with no immature or heterologous elements, regarded as a lipoma.

## Comment

We have presented a female infant with accessory labioscrotal fold bearing a penis-like phallus and perineal lipoma, associated with contralateral renal agenesis and absence of Müllerian/paramesonephric duct structures. Accessory labioscrotal fold is rare. In the first 2 reported female cases,^1,2^ there were no associated anomalies.

In the subsequent third and fourth reported cases,^3^ associated anorectal malformations were present in addition to the perineal lipomas. Three further cases were described from Japan, in which a lipoma occurred within an accessory labioscrotal fold, without additional abnormalities.^4^ In their report, Numajiri et al^4^ also reviewed other reports of perineal lipoma in females without an accessory labioscrotal fold, and none were associated with any genitourinary anomalies.

Our case of accessory labioscrotal fold with perineal lipoma has 2 novel features. First, the association with unilateral renal agenesis and concomitant absence of Müllerian/paramesonephric duct structures, and second, the presence of a phallus with histologic similarity to a penis. In our case, the penis-like phallus lacks a structure analogous to the glans, the distal urethra being lined by urothelium, with no transition to squamous epithelium and the raphe extending to the tip, which lacks a meatus. This suggests that splitting/duplication of the genital tubercle did not occur. The shaft of the phallus has an otherwise close resemblance to penile shaft, with well-formed corpus spongiosum and corpora cavernosa separated by a fibrous septum, with dorsal neurovascular bundle and fibrous layer analogous to the deep fascia. To our knowledge, such close similarity has not been reported in a female case with no evidence of genetic mosaicism. There was no anorectal anomaly/malformation in our case. This is the first report of an association between unilateral renal agenesis and perineal lipoma with accessory labioscrotal fold and accessory phallus.

It has been suggested that accessory phallus in females may form in the absence of virilization as a consequence of abnormal descent of the Müllerian ducts,^5^ and that accessory labioscrotal folds develop when intervening mesenchyme (which later develops into a lipoma) disrupts the continuity of the caudally developing labioscrotal swelling.^1^ Our case has both accessory phallus and a closely apposed accessory labioscrotal fold with a perineal lipoma, and it also has a complete absence of structures derived from the Müllerian/paramesonephric ducts.

The earliest embryological abnormality in our case was the failure of induction of the right metanephric kidney in the fifth week of development, with consequent absence of paramesonephric structures, and development of an accessory phallus without a virilizing influence.^5^ The accessory phallus probably started forming in the seventh week, at around the same time that the labioscrotal swelling formed an accessory labioscrotal fold. The absence of a glans implies that the genital tubercle did not divide and that the accessory phallus is completely derived from mesoderm (except for the skin covering it), as is the perineal lipoma immediately caudal to it. We suggest that an inappropriate mass of mesenchyme caused the formation of an accessory labioscrotal fold,^1^ and in addition to forming the perineal lipoma, its cranial portion developed into a remarkably penis-like phallus secondary to the absent paramesonephric structures. This would represent the collision of 2 rare embryological defects, both of which occur in the absence of virilization and have previously only been reported separately.
